# Preliminary development of a multidimensional positive youth development scale for young rural and urban adolescents in China

**DOI:** 10.1371/journal.pone.0270974

**Published:** 2022-07-28

**Authors:** Ming Wen, Zhi Ye, Danhua Lin, Weidong Wang

**Affiliations:** 1 Department of Sociology, University of Utah, Salt Lake City, UT, United States of America; 2 School of Marxism, Zhejiang Police College, Hangzhou, Zhejiang Province, China; 3 Institute of Developmental Psychology, Beijing Normal University, Beijing, China; 4 National Survey Research Center, Renmin University of China, Beijing, China; National Institutes of Health, UNITED STATES

## Abstract

This study examined the dimensionality, reliability, and validity of a Chinese version of the 5-C Model of positive youth development (PYD), originally developed in the U.S., with a sample of rural and urban young adolescents in China. The Cs represent five youth strengths: competence, confidence, character, caring, and connection. The results of the exploratory factor analyses showed a reasonable data fit with the 5-C Model. The total and subscale scores evinced good internal reliability (α = .7 to .9) and the confirmatory factor analyses confirmed the convergence of the five Cs on a second-order latent factor of PYD, showing adequate goodness of fit (CFI = 0.94; TLI = 0.93; RMSEA = 0.04). Metric and scalar invariance were found across gender. Metric and partial scalar invariance were found for rural-urban groups. Supportive evidence on convergent and discriminatory validity was also found. We conclude that the Chinese version of the 5-C PYD Scale is a reliable and valid instrument, with good construct validity for Chinese young adolescents.

## Introduction

### Conceptualizing and measuring positive youth development (PYD)

The field of psychology has traditionally centered on examining the etiology of mental pathologies and providing therapies and treatments to heal or lessen human mental suffering. In the 1990s, however, a scholarship of positive psychology began to emphasize the assets correlated with psychological well-being, providing substantial evidence on which factors enable positive emotion and what helps individuals, communities, and societies to achieve optimal well-being [1]. Parallel but distinct to the positive psychology movement in research and practice [2], the PYD approach also emerged in the late 1990s and spawned a rapidly growing literature that identifies positive youth qualities and examines the factors and conditions that allow children and adolescents to function optimally.

Guided by developmental systems theories, Eccles and Gootman [3] and Roth and Brooks-Gunn [4] theorized that PYD can be conceptualized into “five Cs”: competence, confidence, character, caring, and connection. According to this framework, a thriving youth should have healthy development in all of these five domains: being *confident* about his/her internal worth; being *competent* at various tasks; having positive bonds with people and institutions (*connection*); exhibiting integrity and respect for societal and cultural rules (*character*); and having a strong sense of sympathy and empathy for others (*caring*) [5].

Drawing from the experiences and views of practitioners, knowledge of scholars, and reviews of the adolescent development literature, Lerner and his collaborators developed the PYD Inventory and launched the 4-H study in 2002, a large-scale longitudinal study [6]. Studies using the 4-H data have supported the 5-C PYD scale, reporting satisfactory psychometric properties and longitudinal measurement invariance across the breadth of the adolescent period of life [2, 7]. However, little is known about whether the 5-C PYD Model also works in other cultural settings.

### Contexts in China

The process of growing up can be stressful, as the child must navigate a range of social, psychological, and biological turbulences on a regular basis. The developmental challenges can be compounded by living in societies experiencing dramatic social changes, like China, which has been undergoing market-oriented reforms and dramatic socio-demographic-cultural transformations for four decades. Societal forces such as the one-child policy implemented from 1979 to 2005 and the massive rural-to-urban migration affecting dozens of millions of rural children make China uniquely situated for studying youth development [8].

The current developmental literature in China generally follows the traditional deficit- or problem-oriented model, paying disproportionately heavy attention to risk factors and antisocial behaviors as problems that need to be fixed, as opposed to focusing on youth assets and resources that need to be recognized and fostered. However, only a minority of young people have a stormy second decade of life [2], and “being problem free is not fully prepared” [1] for youths to enter and flourish in adult society. A strengths-based PYD approach is needed to better understand and promote healthy development among Chinese youths, as it provides a pathway to promote quality of life and mitigate problematic behaviors among Chinese adolescents through facilitating constructive interaction between individuals and their context. The PYD approach can also provide valuable clinical guidance for practitioners and educators to develop and implement intervention programs helping Chinese adolescents from a strength-based perspective.

To stimulate empirical research on PYD in China, there needs to be a valid measure. A comprehensive system of PYD measurement has been developed in Hong Kong, including 80 items that constitute dimensions largely overlapping with the 5-C PYD components and show good psychometric properties [9]. This inventory is not parsimonious, however, especially for national data collection, and no similar psychometric testing for a PYD scale has been conducted using data collected from mainland China.

Evidence indicates that PYD levels are positively associated with adolescents’ school adjustment as measured by school grades and educational aspirations [10, 11] and that higher levels of PYD are also found to be correlated with fewer risky behaviors [12, 13]. We will thus use school adjustment outcomes and risky behaviors to assess the convergent and discriminant validity for our PYD scale, respectively.

### The present study

The present study aims to develop and provide preliminary validation of a PYD instrument for Chinese adolescents, testing the dimensionality, reliability, and validity of the 5-C Model of PYD on a sample of young adolescents currently living in urban and rural areas in China. We first explored whether the PYD scale supported the five-factor construct based on the 5-C Model of PYD developed in the U.S. We then examined the factor structure and group invariances across gender and rural-urban background and investigated the construct validity of PYD scale by examining the whole scale and subscales’ relations with relevant behavioral and educational outcomes.

The PYD scale is designed to be suitable for adolescents aged 12 to 17. In this study, we focused on seventh-graders. The seventh grade is the initial stage of adolescence where dramatic changes in cognitive, emotional, social, and physical domains occur. These changes intensify adolescents’ vulnerability to psychosocial distress and risk behaviors [14, 15]. The seventh grade is a particularly critical stage for Chinese adolescents because they move from elementary school to middle school at this age. They must adapt to new teachers, peers, and school environments and face considerably greater academic pressure than before.

A total of 1,296 seventh-graders were recruited into the study in 2015–2016, of whom 775 were from Beijing and 627 from Anhui. After excluding cases missing the PYD variables, we divided the analytical sample (N = 947) randomly into two groups, one for phase 1 (N = 471) and the other for phase 2 (N = 476).

## Phase 1: Exploratory factor analysis

### Participants and procedure

To capture adolescents from both rural and urban areas, a survey was conducted at two sites sequentially within 6 months: Beijing, as the capital and a large metropolitan area, and a rural county in Anhui Province. The Beijing survey was conducted using a targeted convenient sampling method, recruiting all the seventh-graders from four public schools in the Haidian District, which is the second-largest district in urban Beijing, and currently houses a migrant population of 1.3 million, accounting for 17.8% of Beijing’s total migrants [16].

The Anhui survey was conducted using random sampling. First, a complete list of junior high schools in the county was obtained. Second, a random sample of five schools was selected using probability proportional to size (PPS) sampling based on the number of seventh-graders across schools. Third, all the seventh-graders in the selected schools were recruited into the study. The Anhui sample was thus representative of the county’s seventh-grade population.

After data collection and cleaning, a total sample of 471 seventh-graders participated in the research (*M* age = 13.24, *SD* = 0.68, boys = 55.0%), with 265 participants from Beijing including both urban natives and rural-to-urban migrants (*M* age = 12.99, *SD* = 0.55, boys = 56.6%) and 206 from Anhui including rural natives from migrant (left-behind children; LBC) or non-migrant families (not-left-behind children; not-LBC) (*M* age = 13.57, *SD* = 0.70, boys = 52.9%).

In the current study, we did not obtain parental consent for several reasons. First, parental consent was typically not required by the ethics review board for this type of study in China at the time when we submitted the research protocol. We submitted a request for alteration of informed consent to the Institutional Review Board at the University of Utah. We requested to waive parental consent given that the participants in this study were junior high school students aged approximately 12–14, arguably old enough to give oral consent before they filled out the questionnaire. Moreover, when we explained the consent process to the study participants, we emphasized that they should feel absolutely free to not participate in this study, skip any questions, or stop in the middle of filling out the questionnaire. Second, if written parental consent were a requirement, the investigator would be unable to obtain consent for more than 25% of participants because they were rural children with parents being migrant workers in the cities who would be hard to reach. Third, for our purposes, we believed oral consent would suffice and would make the study more feasible. The Institutional Review Board (IRB) of the University of Utah exempted this study from further review and the Beijing Normal University Ethical Advisory Committee approved the study procedures. A professional teacher or a trained research assistant was present throughout the test administration session. Adequate time was provided for the participants to complete the questionnaire.

### Measures

#### The positive youth development scale

The 5-C Model of PYD was used as theoretical guidance in developing our self-reported survey inventory, which consists of five subscales serving as indicators of the Five Cs. Rather than directly translating the US-based English-language version of the Five Cs scale [17], we independently developed the specific items under each C based on our understanding of the Chinese settings. Table 1 lists all the items on each subscale. The *competence* subscale includes four items and captures overall physical, mental, and cognitive strengths. The *confidence* subscale includes seven items and taps a person’s perceived self-worth. The *character* subscale includes four items and measures integrity and respect for societal and cultural rules. The *caring* subscale includes five items and measures sympathy and compassion. The *connection* subscale includes six items indicating socio-relational resources at school and home and among peers. The total PYD scale includes 26 items.

**Table 1:** Structures and item factor loadings of PYD scale.

Subscale	Items	Factor loading
1. Competence (α = 0.82)	How do you rate your general health?	0.69
	How do you rate your psychological health or spiritual status?	0.69
	People who know me think I’m smart	0.79
	People who know me think my ability is very strong	0.81
2. Confidence (α = 0.72)	Are you confident about what you will do when you grow up?	0.68
	How confident are you about dealing with the problems you will encounter in the future?	0.71
	How confident are you that you can deal with school work as you gradually grow up?	0.70
	How confident are you that you will always someone around you caring about you in the future?	0.72
	How confident are you that you will not encounter much trouble in the future?	0.63
	How confident are you that you will be happy in the future?	0.76
	How confident are you that you can do things that interest you in the future?	0.75
3. Character (α = 0.70)	For me, to make efforts to make the world a better place is very important	0.77
	To spend energy and time to better the lives of others is a very important thing	0.76
	Even when my friends might make fun of me, I will still do what I think is right	0.73
	When I have trouble or make mistakes, I can take responsibility.	0.69
4. Caring (α = 0.86)	It makes me feel uneasy when bad things happen to anyone	0.70
	I feel sorry for the people who cannot have what I have	0.72
	When I see someone being picked on, I feel bad for them	0.83
	I feel sad when I see someone having no friends	0.80
	When I see people suffering or upset, I would feel uneasy	0.84
5. Connection (α = 0.83)	I get a lot of encouragement in school	0.72
	School teachers will help me do what I can do best	0.73
	I have a lot of pleasant conversation with my parents	0.71
	At home, I feel I am important and useful	0.68
	I feel my friends are all very good friends	0.70
	My friends care about me very much	0.75

*N* = 472.

### Data analysis

Exploratory factor analysis (EFA) was sequentially performed to explore the factor structures of PYD using SPSS 22.0. In EFA, factor loadings and Cronbach’s alpha coefficients were used to determine reliability of total scale and subscale scores.

### Results

The subscales for all five Cs exhibited acceptable to good internal reliability (see Table **1**). The 4-item competence subscale had a Cronbach’s alpha coefficient of 0.82 with factor loadings ranging from 0.69 to 0.81. The 7-item confidence subscale had an alpha value of 0.72 with factor loadings from 0.63 to 0.75. The 4-item character subscale had an alpha value of 0.72 with factor loadings from 0.69 to 0.77. The caring subscale had an alpha value of 0.86 with factor loadings from 0.70 to 0.84. The connection subscale had an alpha value of 0.83 with factor loadings ranging from 0.68 to 0.75. These five Cs constituted a second-order latent factor of PYD, which had an alpha value of 0.90 with the factor loadings ranging from 0.52 (caring) to 0.94 (connection).

These findings demonstrate five stable and reliable factors underlying these items, all of which exhibit strong internal reliability. The five factors are conceptually consistent with our hypothesis, tapping into the related but distinct 5-C constructs.

## Phase 2: Confirmatory factor analysis

### Participants

A total sample of 476 students (243 boys, 51.1%) were recruited into the study using the same procedure described in Study 1. The sample had similar characteristics to those in Study 1. Particularly, the mean age of the sample was 13.21 years (*SD* = 0.68), with 284 participants from Beijing including both urban natives and rural-to-urban migrants (*M* age = 12.96, *SD* = 0.54, boys = 50.0%) and 192 from Anhui including rural natives from migrant (left-behind children; LBC) or non-migrant families (not-left-behind children; not-LBC) (*M* age = 13.59, *SD* = 0.69, boys = 52.6%).

### Measures

#### The positive youth development scale

In Study 2, participants were asked to report the levels of PYD using the same scale identified in Study 1. For the sample in the current study, the internal consistency of the subscales was excellent, with 0.72, 0.82, 0.70, 0.86, and 0.83 for *competence*, *confidence*, *character*, *caring*, and *connection*, respectively, and 0.90 for the total scale.

#### Validity indicators

*Educational aspiration* was measured by responses to a question asking what educational level the respondent planned to obtain in the future. The response categories included “below high school,” “high school,” “some college or bachelor’s degree,” and “graduate degree.”

*School grade* was calculated as the average standardized score of curriculum subjects in Chinese, English, and mathematics.

*Risky behaviors* were measured by *smoking* and *drinking* based on responses to the questions “Have you ever smoked?” and “Have you ever had a drink?” with dichotomous responses.

#### Control variables

*Age*, *gender*, *family structure* (1 = intact family; 0 = non-intact family), *parental education*, and *subjective socioeconomic status* (SES) were controlled in the regression analysis testing the construct validity of the PYD scales. Parental education was categorized into four levels: “below high school,” “high school diploma,” “some college or associate degree,” “bachelor’s degree,” and “graduate degree.” Subjective SES was measured by a single question asking “How well off do you think your family is?” Responses to the question included: “not at all well off/ not very well off,” “average,” “quite well off,” and “very well off.”

### Data analysis

Several steps were taken for the data analysis. First, confirmatory factor analysis (CFA) was sequentially performed to test the 5-C Model of PYD using Mplus 7.1, respectively. In CFA, the Robust Maximum Likelihood estimator was used to enhance the interpretability of the results, as it does not require normal distribution of the variables. In addition, to assess goodness of fit for all the models, we used the Root Mean Square Error of Approximation (RMSEA; 0.05 or below indicating excellent fit, 0.05–0.08 reflecting an acceptable fit), Standardized Root Mean Square Residual (0.05 or below indicating excellent fit, 0.05–0.08 indicating an acceptable fit), Comparative Fit Index (CFI; 0.95 or above indicating excellent fit, 0.90–0.95 reflecting an acceptable fit), and Tucker-Lewis Index (TLI; 0.95 or above indicating excellent fit, 0.90–0.95 indicating an acceptable fit).

Second, measurement invariance of the PYD model across gender and rural-urban groups was tested [18]. We tested a sequence of models, beginning with an unconstrained model and progressively introducing equality constraints on parameters [19]. Initially, *configural invariance* was assessed by allowing all parameters to be freely estimated in each group (Model 1). Next, *metric (weak) invariance* was tested by constraining the first-order and second-order factor loadings to be equal in each group. *Scalar (strong) invariance* was subsequently tested by constraining first-order and second-order factor loadings and intercepts to be equal. Lastly, *invariance of residual variance of first-order factors* was tested, in which residual variance of first-order factors and of the measured variables were constrained to be equal across groups. Each model represented a more restricted parameterization than the previous one, and Δ*χ*^*2*^ and ΔCFI were used to determine whether the data supported the measurement invariance of the nested models [20].

Third, construct validity of the PYD scale was tested by performing multiple ordinary least square (OLS) or logistic regression modeling between the PYD scale and four indicators of adolescent development.

### Results

#### Confirmatory factor analysis

**Fig 1:**
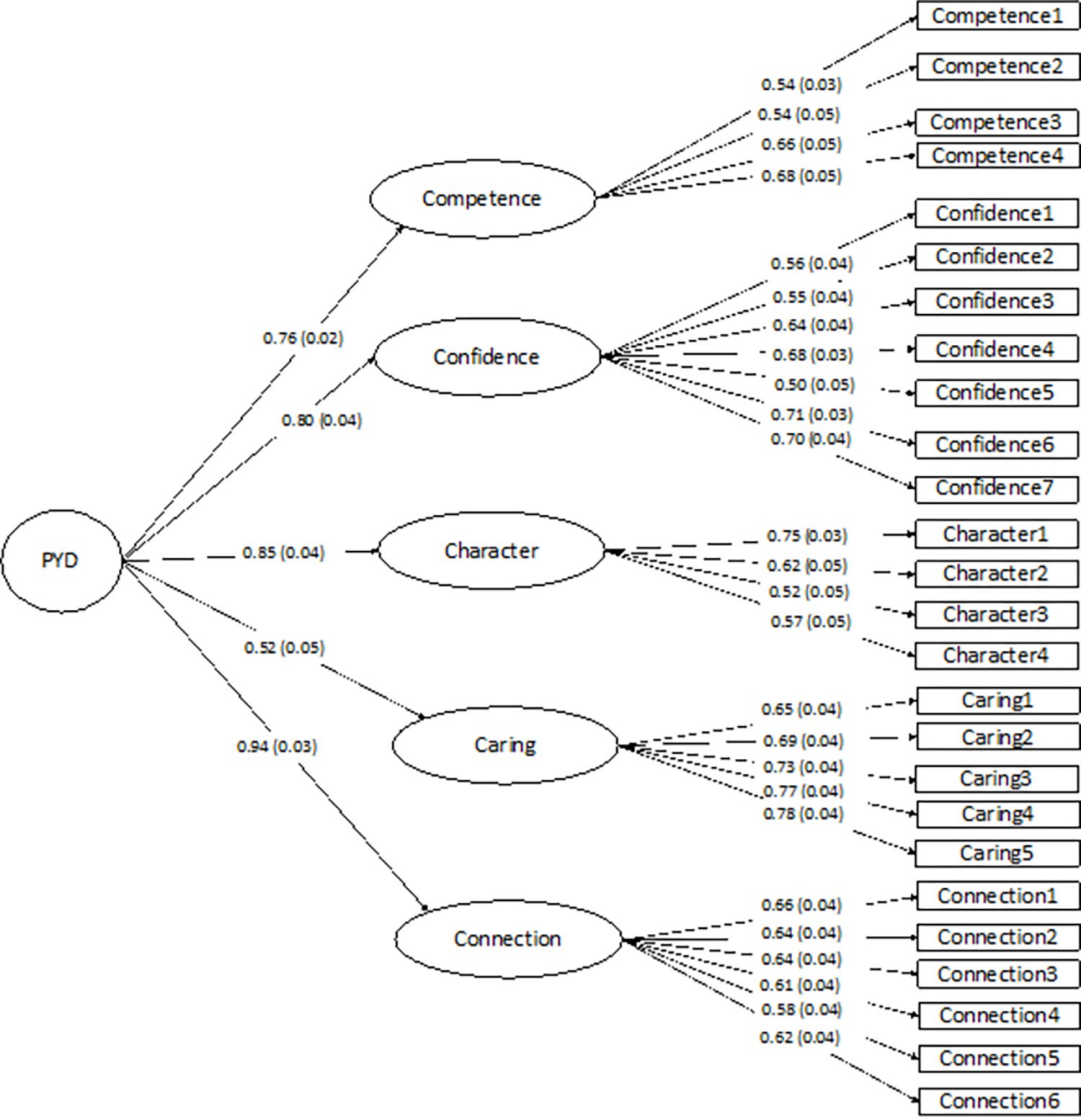
PYD measurement model with standardized parameter estimates.

CFA was conducted using a sample size of 476 cases to examine the factor structure of the PYD scale with the five Cs as first-order latent factors that loaded on a second-order latent factor of PYD using a robust maximum-likelihood method (see Fig 1). The CFA statistics indicated good fitness (*χ*^*2*^ = 515.31; *df* = 286; CFI = 0.94; TLI = 0.93; RMSEA = 0.04).

#### Measurement invariance

Table 2 presents the results from testing the measurement invariance across gender using multiple-group CFAs. Configural invariance was supported by the data according to a series of model fitness statistics (*χ*^*2*^ = 834.054, *df* = 563, CFI = 0.932, TLI = 0.921, RMSEA = 0.045) (Model 1). Next, no significant differences were observed between the configural model and the metric invariance models which constrained the first-order and second-order factor loadings to be equal (Model 2 and Model 3). In addition, results from Model 4 and Model 5 further showed that there was no significant measurement difference across gender given Δ CFIs < 0.01 in both models. These results suggest that the measurement invariance across gender was observed. In other words, the five Cs items did measure the same PYD construct for boys and girls.

**Table 2:** Testing invariance of PYD scale structure by gender.

Model	*χ* ^*2*^	*df*	CFI	TLI	RMSEA	Model comparison	*Δχ* ^*2*^	*Δ* CFI
(1) Configural model	834.054	563	0.932	0.921	0.045	-	-	-
(2) Invariance of first- and second-order factor loadings	853.041	584	0.932	0.925	0.044	2 vs. 1	18.987	0.000
(3) Invariance of first- and second-order factor loadings	854.384	588	0.933	0.926	0.044	3 vs. 2	1.343	0.001
(4) Invariance of intercepts of measured variables	908.442	614	0.926	0.922	0.045	4 vs. 3	54.058***	-0.007
(5) Invariance of residual variances of first-order factors	926.299	640	0.928	0.927	0.043	5 vs. 4	17.857	0.002

*N* = 475

Next, the Five Cs of PYD scale was also tested for factorial invariance across four rural-urban youth groups. The results indicated metric invariance across the four groups (see Table 3, Model 2, and Model 3). However, when we examined scalar invariance, the intercepts of four items of *competence* and seven items of *confidence* were found to differ significantly across the groups. Therefore, the constraints on the intercepts of competence and confidence were freed and partial scalar invariance was observed in Model 4a, as indicated by the significant *Δχ*^*2*^ and insignificant *Δ* CFI when compared to Model 3. The difference between Model 5 and Model 4a was also significant in *Δχ*^*2*^ but not in Δ CFI. These results provide some evidence to show that partial but not complete measurement invariance across the rural-urban groups was observed.

**Table 3:** Testing invariance of PYD scale structure by rural-urban status.

Model	*χ* ^*2*^	*df*	CFI	TLI	RMSEA	Model comparison	*Δχ* ^*2*^	*Δ* CFI
(1) Configural model	1655.885	1118	0.884	0.865	0.064	-	-	-
(2) Invariance of first-order factor loadings	1731.860	1181	0.881	0.869	0.063	2 vs. 1	75.975	-0.003
(3) Invariance of first- and second-order factor loadings	1740.226	1193	0.882	0.871	0.062	3 vs. 2	8.366	0.001
(4) Invariance of intercepts of measured variables	1868.692	1271	0.871	0.868	0.063	4 vs 3	128.466***	-0.011
(4a) Invariance of intercepts of measured variables- 11 intercepts freed	1822.514	1238	0.874	0.867	0.063	4a vs. 3	82.288***	-0.008
(5) Invariance of residual variances of first-order factors	1938.630	1316	0.865	0.867	0.063	5 vs 4a	116.116**	-0.009

*N* = 475.

#### Convergent/Discriminant validity

The association between the total PYD scale and prosocial outcomes was examined using OLS regression. After controlling for sociodemographic factors, the total PYD scale was significantly and positively associated with educational aspiration (coefficient = 0.16; *p* < 0.001) and school grade (coefficient = 0.09; *p* < 0.05), showing *convergent validity* (see Table 4). All five Cs showed positive associations with the two outcomes, while the effects of confidence and caring were not significant.

**Table 4:** OLS regression coefficients of total PYD scale on educational aspiration and school grade.

	Educational aspiration		School grade	
	*ß*	*ΔR* ^*2*^	*ß*	*ΔR* ^*2*^
Total PYD scale	0.16***	0.02***	0.09*	0.01*
Competence	0.22***	0.04***	0.13**	0.01**
Confidence	0.10	0.01*	0.01	0.00
Character	0.12**	0.01**	0.05	0.00
Caring	0.02	0.00	0.03	0.00
Connection	0.15***	0.02***	0.11**	0.01**

*N* = 475; Socio-demographic factors controlled; Standardized coefficients reported

*p < 0.05

**p < 0.01

***p < 0.001.

The relationship between total PYD scale and risky behaviors was tested using logistic regression (see Table 5). After controlling for sociodemographic factors, competence (OR = 0.38; *p* < 0.05) and character (OR = 0.45; *p* < 0.05) were both negatively associated with ever smoking. As for drinking behavior, total PYD showed a significant association (OR = 0.57; *p* < 0.05). Specifically, one SD increase in PYD was linked to 75% lower odds of drinking for the seventh-graders in the sample.

**Table 5:** Logistic regression odds ratios of total PYD scale on smoking and drinking.

	Smoking behavior		Drinking behavior	
	OR	95% CI	OR	95% CI
Total PYD scale	0.42	0.15–1.20	0.57*	0.37–0.89
Competence	0.38*	0.16–0.89	0.72^†^	0.52–1.01
Confidence	0.85	0.36–2.01	0.70*	0.50–0.99
Character	0.45*	0.20–1.00	1.23	0.89–1.69
Caring	0.93	0.52–1.67	0.96	0.76–1.21
Connection	0.63	0.29–1.33	0.61***	0.45–0.82

*N* = 475; Socio-demographic factors controlled; Odds Ratios (OR) presented

^†^ *p* < 0.10

*p < 0.05

**p < 0.01

***p < 0.001.

## Discussion

This study examined the dimensionality, reliability, and validity of the 5-C Model of PYD with a sample of young adolescents distinguished by rural-urban origin and residence. We performed exploratory and confirmatory analyses and found that the conceptual dimensions of the 5-C Model of PYD [6] were generally supported in this sample. The total and subscale scores evinced acceptable to very good internal reliability, with these scales’ alpha coefficients ranging from 0.70 to 0.90. The confirmatory factor analyses confirmed the convergence of these five Cs on a second-order latent factor of PYD, showing adequate goodness of fit (***χ***^***2***^ = 515.31; *df* = 286; CFI = 0.94; TLI = 0.93; RMSEA = 0.04).

Among the five Cs, competence, confidence, character, and connection were all tightly clustered, but caring fit less well, with a factor loading of 0.52—considerably lower than the factor loadings of the other four Cs, ranging from 0.76 on competence to 0.94 on connection. This is consistent with its smaller correlation coefficients with the other Cs: coefficients for caring ranged from 0.17 to 0.38 compared to those among the other Cs, which ranged from 0.47 to 0.57. Caring was also the least correlated with the total PYD scale, with a correlation coefficient of 0.64, compared to the other four coefficients being greater than 0.80. This finding is consistent with results from U.S.-based studies. For example, the factor loading of caring on the total PYD scale based on the five Cs was 0.43 to 0.46 among the fifth-graders in the 4-H study [7]. In a longitudinal study of eighth-, ninth-, and tenth-graders using the 4-H data [5], caring also exhibited the lowest factor loadings, ranging from 0.53 to 0.63, compared to those of the other Cs for each grade. Perhaps it can be speculated that caring may not always be adaptive, because high levels of caring often signal high levels of concern and sensitivity to others’ thoughts and feelings. Excessive caring might come at social-emotional costs, given its positive association with anxiety and depressive feelings for adolescents [21, 22]. This phenomenon is not unique to Chinese culture, considering that consistent findings from U.S.-based studies also show a poorer fit of caring in the 5-C PYD Model [5, 7]. In any event, more research needs to be done to better understand the co-occurrence of different desirable characteristics that can be encapsulated into the PYD framework.

The present work also tested the factorial invariance across gender and rural-urban groups. Metric and scalar invariance were found across gender, suggesting that the five-factor PYD scale should function similarly across boys and girls. Metric and partial scalar invariance were found for rural-urban groups. Specifically, scoring variation was found on competence and confidence items. This suggests that caution is needed in interpreting results on rural-urban group differences in competence and confidence if measured using these items. More qualitative and quantitative work is needed to explore the conceptual components of the PYD framework and develop an effective inventory that is valid across different socio-demographic groups.

In terms of construct validity, convergent validity was confirmed by the positive associations of the total PYD scale with positive outcomes like educational aspiration and school grade, and discriminatory validity was supported by the negative link between the total PYD scale and drinking behavior. Discriminatory validity was not found for smoking behavior, primarily due to lacking an effect for confidence, caring, and connection. Smoking prevalence was about 3% in this sample, which may partly explain the low ability of the PYD total scale in discriminating smokers from nonsmokers. Among the five Cs, competence showed the most consistent associations with the four known developmental outcomes used to examine the PYD scale’s construct validity. By contrast, caring was not linked to any of the four outcomes, and confidence was also not significant for three out of the four outcomes. Clearly, some Cs have better predictive power for known developmental outcomes than others. A recent study conducted among Irish adolescents reported anomaly findings on confidence, showing that higher scores on the confidence subscale were related to lower contribution and higher rates of risky behaviors [17]. The authors speculated that confidence was positively associated with narcissism, which has been found to predict high levels of conduct problems and internalizing problems in young people [23]. It is also intriguing that caring did not fit the PYD model as well as the other four Cs and was not a significant covariate for any of the four developmental outcomes used to assess PYD’s construct validity. The caring subscale consisted of five items showing strong reliability with an alpha value of 0.86 and factor loadings all above 0.70. It is possible that the correspondence between caring and the other desirable qualities in youth is not as strong as that among the other Cs. Despite this, compassion is one of the most highly desirable traits that a thriving youth is expected to have. It would be interesting to see how different Cs differ in predicting different aspects of future contributions.

In sum, this study found the 5-C Model of PYD to be an adequate structural model to depict youth thriving in early adolescence in China, and the five-factor PYD scale appeared to be a reliable and valid measurement tool. It is useful to note that the PYD inventory used in this study had a total of 26 items measuring all five Cs, which was considerably less cumbersome than PYD inventories examined in previous research [7, 9, 17, 24]. On the positive side, this short list of items exhibited good reliability and validity and can be easily incorporated into new surveys or added to ongoing prospective surveys. On the other hand, more items may be needed to capture the full spectrum of a construct’s components. For example, our competence subscale only captured mental and physical health, cognitive capacity, and general ability, while physical competence was not specifically tapped. Adding a physical competence subscale would presumably enhance the precision of measuring the competence construct, as was done in a U.S. study [7], but it would also make the questionnaire longer. There is always a tradeoff between the quantity of questions and the quality of answers.

Two study limitations are noteworthy. First, this was a cross-sectional study and only focused on seventh-graders sampled from Beijing and a rural county in Anhui Province. The predictive validity cannot be assessed, and the generalizability of the findings to other age groups or other areas should not be assumed. National and longitudinal data are needed to test the structural invariance across different stages of adolescence and assess the predictive power of the PYD measures for future developmental outcomes in representative samples. Second, measurement invariance was not achieved across different rural-urban youth groups. More qualitative and quantitative work must be done to conceptualize PYD components and develop an inventory featuring rural-urban invariance among Chinese adolescents.

Despite these limitations, this work moves the field of child development in China closer to the PYD perspective and strengths-based approach by establishing the existence of a reliable and valid measure of PYD among Chinese early adolescents living in rural and urban areas. The present study was designed to be the first step in depicting youth thriving under the guidance of the 5C-PYD framework, offering an effective measurement tool for evaluating the levels and tracking the developmental trajectories of PYD, and providing a reliable way to investigate the positive outcomes of youth-serving programs seeking to promote PYD among adolescents in a Chinese context. That said, the current study focuses mainly on early adolescents, and future research is needed to further conceptualize PYD components and develop PYD inventories that are invariant across a broader range of sociodemographic groups to better understand patterns and sources of PYD disparities and inform policies and programs aimed at promoting youth thriving in China.

## Supporting information

S1 Data (SAV)

S2 Data (SAV)
